# Identifying the long-term survival beneficiary of preoperative radiotherapy for rectal cancer in the TME era

**DOI:** 10.1038/s41598-022-08541-1

**Published:** 2022-03-17

**Authors:** Lei Wang, Xiaohong Zhong, Huaqin Lin, Xueqing Zhang, Lingdong Shao, Gang Chen, Junxin Wu

**Affiliations:** 1 Department of Radiation Oncology, College of Clinical Medicine for Oncology, Fujian Medical University & Fujian Cancer Hospital, 420 Fuma Rd, Jin’an District, Fuzhou, 350011 Fujian China; 2 Department of Pathology, College of Clinical Medicine for Oncology, Fujian Medical University & Fujian Cancer Hospital, 420 Fuma Rd, Jin’an District, Fuzhou, 350011 Fujian China

**Keywords:** Cancer therapy, Gastrointestinal cancer

## Abstract

This study was to verify the long-term survival efficacy of preoperative radiotherapy (preRT) for locally advanced rectal cancer (LARC) patients and identify potential long-term survival beneficiary. Using the Surveillance, Epidemiology, and End Results (SEER) database, 7582 LARC patients were eligible for this study between 2011 and 2015 including 6066 received preRT and 1516 received surgery alone. Initial results showed that preRT prolonged the median overall survival (OS) of LARC patients (HR 0.86, 95% CI 0.75–0.98, P < 0.05), and subgroup analysis revealed that patients with age > 65 years, stage III, T3, T4, N2, tumor size > 5 cm, tumor deposits, and lymph nodes dissection (LND) ≥ 12 would benefit more from preRT (all P < 0.05). A prognostic predicting nomogram was constructed using the independent risk factors of OS identified by multivariate Cox analysis (all P < 0.05), which exhibited better prediction of OS than the 8th American Joint Cancer Committee staging system on colorectal cancer. According to the current nomogram, patients in the high-risk subgroup had a shorter median OS than low-risk subgroup (HR 2.62, 95% CI 2.25–3.04, P < 0.001), and preRT could benefit more high-risk patients rather than low-risk patients. Hence, we concluded that preRT might bring long-term survival benefits to LARC patients, especially those with high risk.

## Introduction

Colorectal cancer is the third most common malignant cancers worldwide and the second leading cause of cancer mortality globally^1^. In the United States, 43,340 new patients were diagnosed with rectal cancer, including 25,960 men and 17,380 women in 2020^2^. Nonetheless, the mortality from CRC has decreased steadily from 1990 to 2007^3^, and is currently down by approximately 50% from the peak mortality rates^2^, perhaps owing to earlier diagnoses by screening and comprehensive treatment.

Preoperative radiotherapy (preRT), either long-course chemoradiotherapy (LCRT) or short-course radiotherapy (SCRT), followed by radical surgery has been the standard treatment for locally advanced rectal cancer (LARC)^4^. The reasons are mainly due to its advantages on downstaging, pathological complete response (pCR), sphincter preservation and decrease of local recurrence^5–7^. And elevated pCR rate has been observed in recent trials of RAPIDO^5^, PRODIGE-23^8^, and STELLAR^9^ with the intensified neoadjuvant strategies. However, the long-term survival benefit has rarely been established^6–8^. The advent of the total mesorectal excision (TME) has decreased the local recurrence rate to approximately 9%^10^, and treatment failure primarily presents as distant metastasis rather than local recurrence^11,12^.

In 2019, a retrospective study based on the Surveillance, Epidemiology, and End Results (SEER) database verified the survival benefit of preRT in 49,439 patients, but the study had critical limitations^13^. First, the study span ranging from 1988 to 2011 was too long to avoid confounding factors: surgical technique evolved from local excision to TME, radiation therapy progressed from convention radiotherapy to intensity-modulated radiation therapy, and the timeframe of intensive chemotherapy shifted from postoperative to preoperative. Therefore, the effects of preRT on the long-term survival benefits deserves to be re-evaluated.

This study analyzed patients from the SEER database diagnosed between 2011 and 2015 to re-evaluate the clinical value of preRT in the TME era and identify candidates who may benefit from preRT in long-term survival. In addition, we also summarized phase III randomized clinical trials (RCTs) to elucidate long-term survival failure reasons.

## Methods

### Ethics statement

We gained an official permit to access the research data (ID: 12598-Nov2019), and all the analysis in the current study was conducted under the rules of SEER database. The ethical committee waved away the formal ethical approval, mainly due to that all the data was derived from public database and individual information was anonymous.

### Data source and patient selection

The SEER database was screened for patients diagnosed with rectal cancer between 2011 and 2015 using SEER*Stat software (version 8.3.8) with the International Classification of Diseases for Oncology 3rd Edition (ICD-O-3) code: C20.9 Rectum, NOS. Rectal cancer cases were retrieved based on the following: adenocarcinoma (ICD-O-3 codes: 8140/3, 8144/3, 8210/3, 8221/3, 8255/3, 8263/3, and 8572/3); stage T3–4 or N+ and M0 tumors (derived from derived AJCC T, 7th ed (2010–2015), derived AJCC N, 7th ed (2010–2015) and derived AJCC M, 7th ed (2010–2015)); received surgery, including lymphadenectomy (derived from reason no cancer-directed surgery, and regional nodes examined examination (1988+)); and underwent chemotherapy (derived from chemotherapy recode). Cases were excluded if there were multiple primary tumors or no preRT records. All patients underwent active follow-ups. The T/N classification was re-staged according to the 8th edition of the American Joint Committee on Cancer (AJCC) staging system with the following codes: derived AJCC T, 7th ed. (2010–2015); derived AJCC N, 7th ed. (2010–2015); CS extension (2004–2015); CS lymph nodes (2004–2015); and CS site-specific factor 4 (2004+ varying by schema). The endpoint was overall survival (OS), which was extracted directly from the database as months.

### Variable definitions and stratification

The data were collected and re-categorized as follows: age at diagnosis (≤ 65 years, > 65 years), sex (male, female), insurance (no, yes), serum carcinoembryonic antigen (CEA) level (≤ 5 ng/mL, > 5 ng/mL), stage (II, III), T stage (T0/T1/T2, T3, T4), N stage (N0, N1, N2), tumor differentiation (I/II, III/IV), tumor size (≤ 3 cm, 3–5 cm, > 5 cm), tumor deposits (negative, positive), perineural invasion status (absent, present), number of dissected lymph nodes (LND) (< 12, ≥ 12), preRT (no, yes), and survival (months).

### Statistical analyses

Continuous variables were re-defined as categorical variables and presented as frequencies and percentages. Kaplan–Meier curves were plotted to compare the survival difference between patients who received preRT and those who received surgery alone (presented with hazard ratios [HR] and 95% confidence intervals [CI]). Subgroup analysis among patients who received preRT or not were further stratified by variable and plotted using a forest map.

The entire data set was randomly divided into training and validation sets at a 6:4 ratio and compared using the chi-square test or Fisher’s test. A nomogram was established based on the multivariate analysis results, which integrated all of the independent prognostic factors. A calibration plot was constructed to evaluate the calibration of the nomogram. Harrell’s concordance index (C-index) and the area under the receiver operating characteristic curve (AUC) were used to assess the predictive outcome performance of the nomogram and the outcomes. The clinical utility of the nomogram was also evaluated using decision curve analysis (DCA), which included the continuous risk of the probability threshold (x-axis) and the net benefit (y-axis). The nomogram was also compared with the 8th AJCC staging system.

Statistical tests were conducted using RStudio (version 1.3.1073), including the xlsx, table1, survminer, survival, forestplot, rms, nomogramFormula, timeROC, stdca, and survcomp packages. All statistical tests were two-tailed, and statistical significance was set to P < 0.05.

**Table 1:** Demographic and tumor characteristics of 7582 rectal cancer patients.

Character	n	%
Sample size	7582	100.0
**Age**		
≤ 65 years	5421	71.5
> 65 years	2161	28.5
**Sex**		
Male	4649	61.3
Female	2933	38.7
**Insurance**		
No	299	3.9
Yes	7200	95.0
Unknown	83	1.1
**CEA**		
≤ 5 ng/mL	3047	40.2
> 5 ng/mL	2300	30.3
Unknown	2235	29.5
**Stage**		
II	2752	36.3
III	4817	63.5
Unknown	13	0.2
**T stage**		
T0/T1/T2	670	8.8
T3	6065	80.0
T4	804	10.6
Unknown	43	0.6
**N stage**		
N0	2671	35.2
N1	3787	49.9
N2	1111	14.7
Unknown	13	0.2
**Tumor differentiation**		
Grade I/II	5961	78.6
Grade III/IV	827	10.9
Unknown	794	10.5
**Tumor size**		
≤ 3 cm	1811	23.9
3–5 cm	2570	33.9
> 5 cm	2220	29.3
Unknown	981	12.9
**Tumor deposits**		
Negative	6094	80.3
Positive	961	12.7
Unknown	527	7.0
**Perineural invasion**		
Absent	5876	77.5
Present	903	11.9
Unknown	803	10.6
**Number of LND**		
< 12	1992	26.3
≥ 12	5577	73.5
Unknown	13	0.2
**Preoperative radiotherapy**		
No	1516	20.0
Yes	6066	80.0

*CEA* carcinoembryonic antigen, *LND* dissected lymph nodes.

## Results

### Patient characteristics

There were 7582 eligible patients according to the predesigned flow chart (Supplementary Fig. S1), and their baseline characteristics are presented in Table 1; 6066 patients (80.0%) received preRT, and 1516 patients (20.0%) received surgery alone. There were 2752 patients (36.3%) at stage II and 4817 (63.5%) at stage III according to the 8th AJCC staging system. In addition, 73.5% of patients had ≥ 12 LND.

### The effect of preoperative radiotherapy on LARC patients’ long-term survival

**Figure 1:**
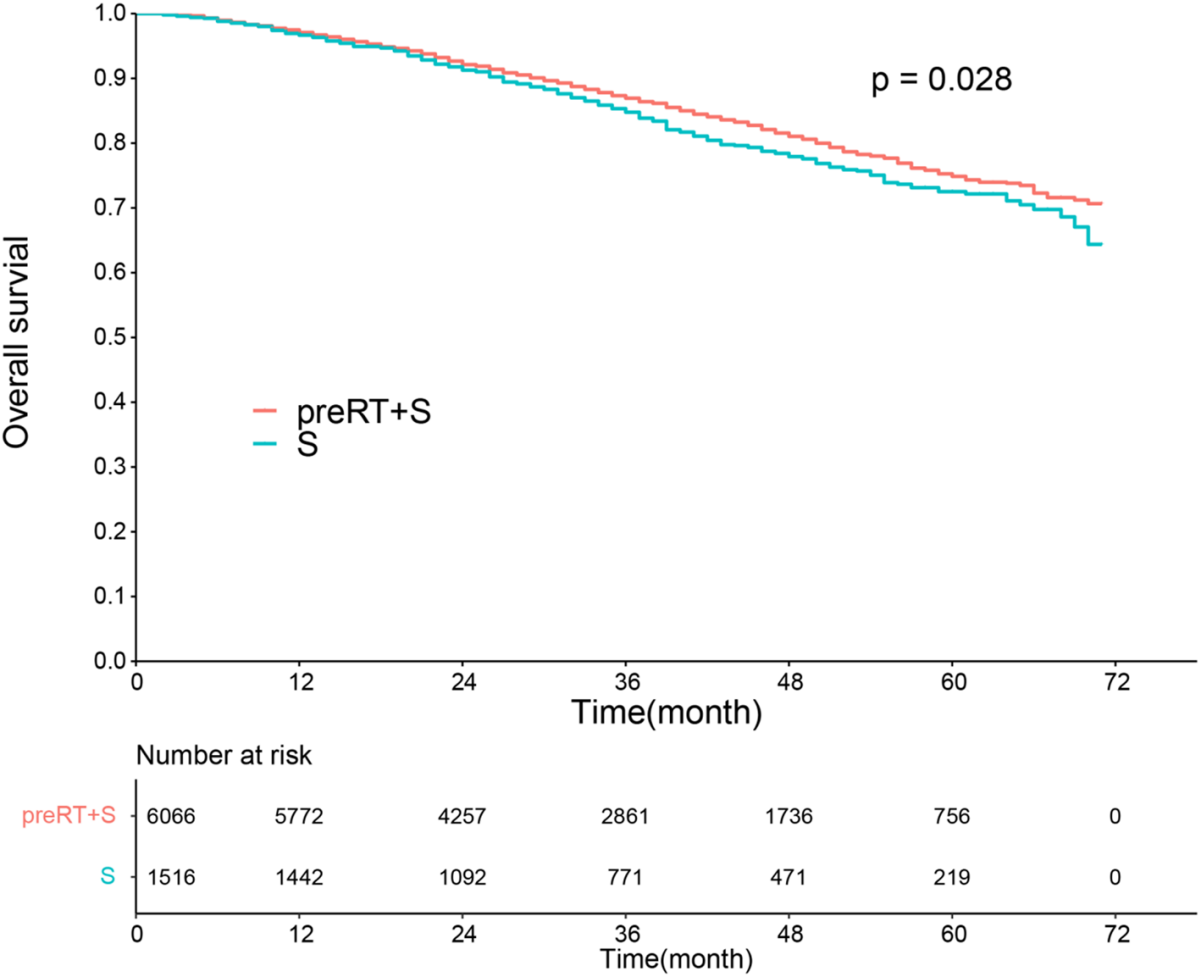
Kaplan–Meier analysis of overall survival according to preoperative radiotherapy (preRT). *S* surgery.

For the entire cohort, Kaplan–Meier survival curve showed that the pooled HR for the median OS favored preRT instead of surgery alone (HR 0.86, 95% CI 0.75–0.98, P = 0.028, Fig. 1). The 3- and 5-year survival rates with or without preRT were all over 70% (86.9% vs. 74.9%; 84.8% vs. 72.5%; respectively). Multivariate Cox regression analysis showed that preRT was not an independent risk factor for OS (HR 0.97, 95% CI 0.80–1.17, P = 0.728).

### Subgroup analysis of OS stratified by risk factor

**Figure 2:**
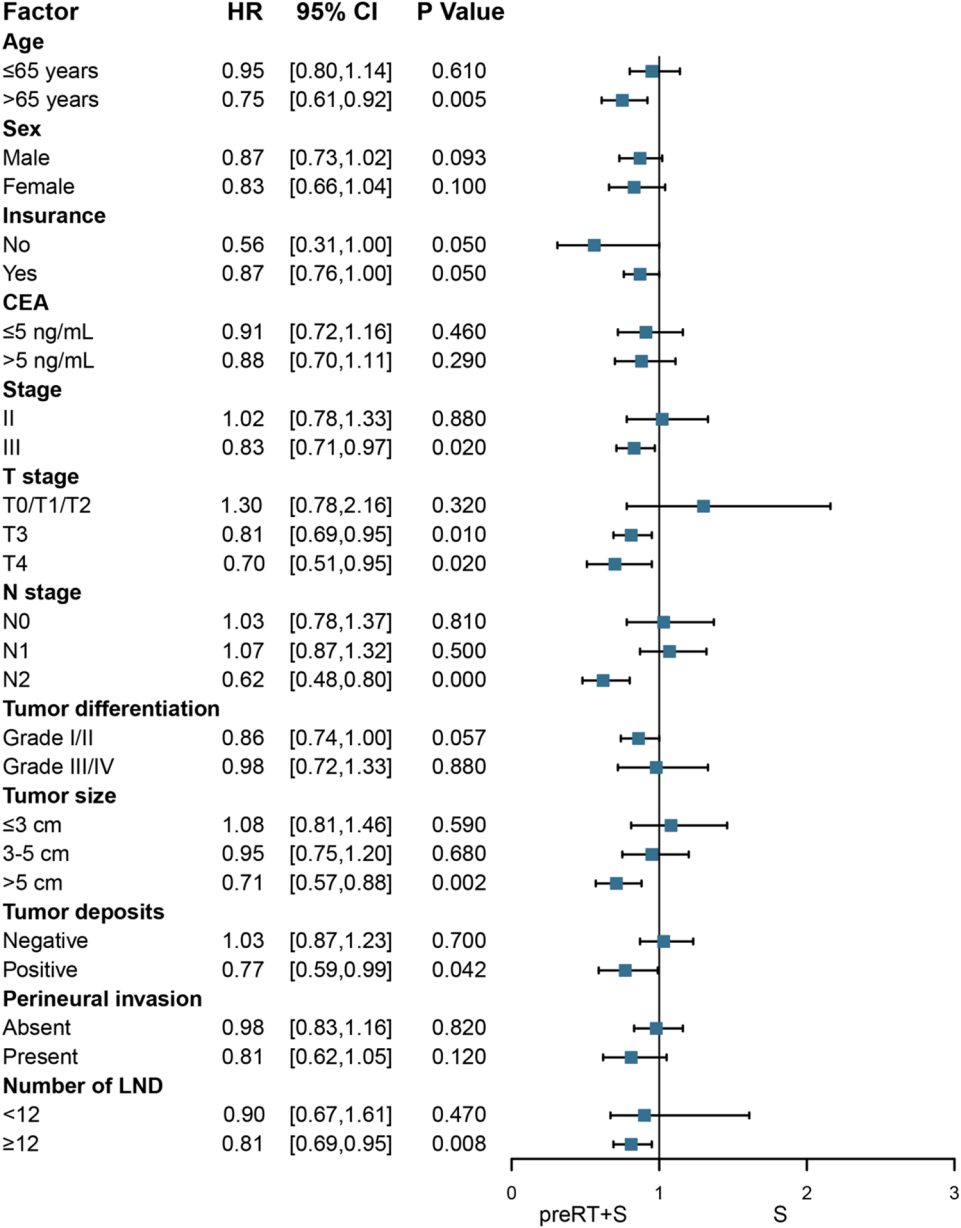
Subgroup analysis of OS stratified by risk factor. *CEA* carcinoembryonic antigen, *LND* dissected lymph nodes, *preRT* preoperative radiotherapy, *S* surgery, *HR* hazard ratio, *CI* confidence interval.

Using the entire cohort, subgroup analysis showed that patients with the following risk factors benefited from preRT regarding OS (Fig. 2): aged > 65 years (HR 0.75, 95% CI 0.61–0.92, P = 0.005), stage III (HR 0.83, 95% CI 0.71–0.97, P = 0.020), T3 (HR 0.81, 95% CI 0.69–0.95, P = 0.010), T4 (HR 0.70, 95% CI 0.51–0.95, P = 0.020), N2 (HR 0.62, 95% CI 0.48–0.80, P < 0.001), tumor size > 5 cm (HR 0.71, 95% CI 0.57–0.88, P = 0.002), tumor deposits (HR 0.77, 95% CI 0.59–0.99, P = 0.042), and LND ≥ 12 (HR 0.81, 95% CI 0.69–0.95, P = 0.008).

### Nomogram construction for LARC prognosis

**Figure 3:**
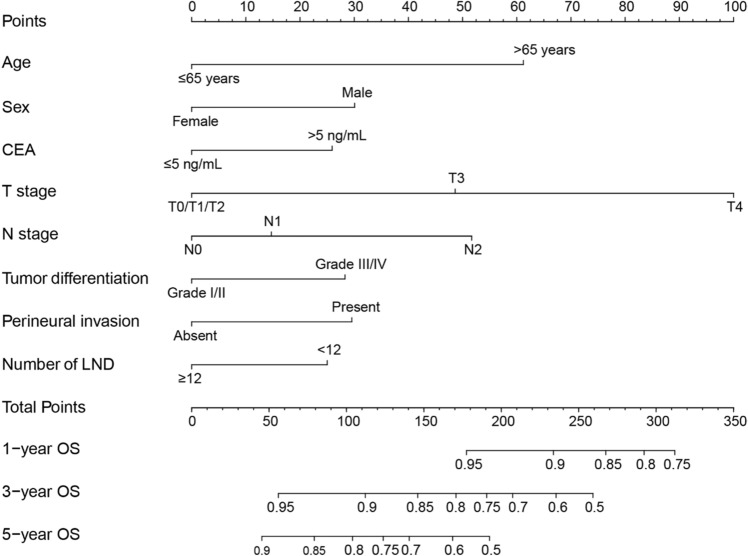
Locally advanced rectal cancer survival nomogram. *OS* overall survival, *CEA* carcinoembryonic antigen, *LND* dissected lymph nodes.

The entire cohort was randomly divided into training and validation sets (6:4 ratio, Supplementary Table S1). The univariate and multivariate Cox regression results for the training set are presented in Table 2. Age > 65 years, CEA > 5 ng/mL, T3, T4, N2, tumor differentiation III/IV, and perineural invasion were independent risk factors for OS (all P < 0.05), while female and LND ≥ 12 were independent protective factors for OS (both P < 0.05). A prognostic nomogram was developed based on the multivariate Cox regression results to predict the 1-, 3-, and 5-year survival rates (Fig. 3).

**Table 2:** Univariate and multivariate analysis of prognostic factors associated with overall survival (training set).

Variable	Univariate analysis		Multivariate analysis	
	HR (95% CI)	P value	HR (95% CI)	P value
**Age**				
≤ 65 years	Reference	–	Reference	–
> 65 years	1.89 (1.63, 2.19)	0.000	2.25 (1.82, 2.78)	0.000
**Sex**				
Male	Reference	–	Reference	–
Female	0.73 (0.63, 0.85)	0.000	0.65 (0.52, 0.82)	0.000
**Insurance**				
No	Reference	–		
Yes	0.77 (0.55, 1.07)	0.116		
**CEA**				
≤ 5 ng/mL	Reference	–	Reference	–
> 5 ng/mL	1.65 (1.38, 1.97)	0.000	1.40 (1.13, 1.74)	0.002
**T stage**				
T0/T1/T2	Reference	–	Reference	–
T3	1.61 (1.17, 2.22)	0.004	1.80 (1.05, 3.09)	0.032
T4	3.51 (2.46, 5.02)	0.000	3.58 (1.99, 6.43)	0.000
**N stage**				
N0	Reference	–	Reference	–
N1	1.25 (1.06, 1.48)	0.008	1.16 (0.89, 1.51)	0.266
N2	1.87 (1.52, 2.30)	0.000	2.02 (1.47, 2.77)	0.000
**Tumor differentiation**				
Grade I/II	Reference	–	Reference	–
Grade III/IV	1.63 (1.35, 1.98)	0.000	1.45 (1.11, 1.88)	0.006
**Tumor size**				
≤ 3 cm	Reference	–	Reference	–
3–5 cm	1.10 (0.89, 1.35)	0.375	0.90 (0.68, 1.18)	0.451
> 5 cm	1.53 (1.25, 1.86)	0.000	1.29 (0.98, 1.69)	0.066
**Tumor deposits**				
Negative	Reference	–	Reference	–
Positive	1.99 (1.66, 2.39)	0.000	1.30 (0.98, 1.71)	0.066
**Perineural invasion**				
Absent	Reference	–	Reference	–
Present	1.98 (1.64, 2.38)	0.000	1.35 (1.03, 1.78)	0.030
**Number of LND**				
< 12	Reference	–	Reference	–
≥ 12	0.79 (0.68, 0.93)	0.004	0.68 (0.54, 0.85)	0.001
**Preoperative radiotherapy**				
No	Reference	–	Reference	–
Yes	0.86 (0.72, 1.02)	0.081	1.00 (0.77, 1.28)	0.972

*CEA* carcinoembryonic antigen, *LND* dissected lymph nodes, *HR* hazard ratio, *CI* confidence interval.

### Nomogram predictive performance

The C-index of the nomogram was 0.70 in the training set (95% CI 0.67–0.72) and 0.67 in the validation set (95% CI 0.63–0.71), which were higher than the 8th AJCC staging system (training set: 0.63, 95% CI 0.60–0.67, P < 0.001; validation set: 0.61, 95% CI 0.56–0.66, P = 0.005; Table 3). The nomogram also had better discrimination compared to the 8th AJCC staging system using time-dependent AUC analysis. The 1-, 3-, and 5-year AUC with 95% CIs for different models are presented in Table 3. Additionally, there was good consistency between the observed and the predicted outcomes of the nomogram regarding 3- and 5-year OS in the training and validation sets using calibration plots (Supplementary Fig. S2). DCA also showed that the nomogram had better net benefits at the 5-year than the 8th AJCC staging system in the training (Supplementary Fig. S3A) and validation sets (Supplementary Fig. S3B).

**Table 3:** Comparison of time-dependent AUC and C-index between the nomogram and the 8th AJCC staging system.

	Training set			Validation set		
	Nomogram	8th AJCC	P-value	Nomogram	8th AJCC	P-value
1-year AUC (95% CI)	0.71 (0.65, 0.77)	0.57 (0.50, 0.64)	0.000	0.74 (0.66, 0.81)	0.58 (0.49, 0.66)	0.000
3-year AUC (95% CI)	0.71 (0.68, 0.75)	0.63 (0.59, 0.67)	0.000	0.67 (0.63, 0.71)	0.58 (0.54, 0.63)	0.000
5-year AUC (95% CI)	0.69 (0.64, 0.73)	0.61 (0.56, 0.65)	0.001	0.68 (0.63, 0.73)	0.61 (0.56, 0.66)	0.040
C-index (95% CI)	0.70 (0.67, 0.72)	0.63 (0.60, 0.67)	0.000	0.67 (0.63, 0.71)	0.61 (0.56, 0.66)	0.005

*AUC* area under the receiver operating characteristic curve, *CI* confidence interval, *AJCC* American Joint Committee on Cancer.

### Clinical application of the nomogram

**Figure 4:**
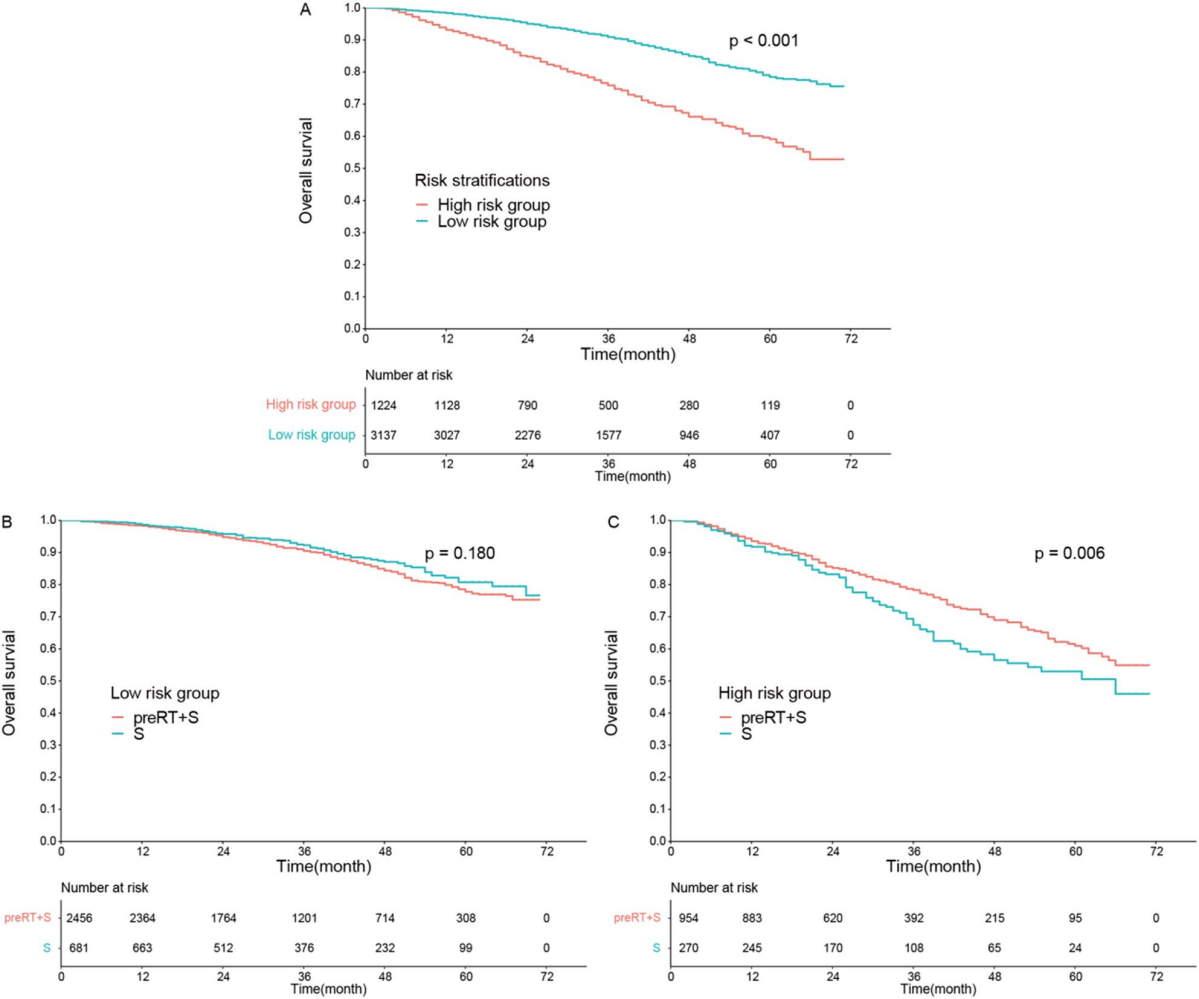
Kaplan–Meier analysis of overall survival according to (**A**) risk stratification; (**B**) preoperative radiotherapy (preRT) for low‐risk patients and (**C**) preoperative radiotherapy for high‐risk patients. *S* surgery.

Each patient’s total score was determined based on the nomogram. The median total score for the entire dataset was 129 (range 24–296). A score of 158 was set as the cut-off value to divide patients into high- and low-risk groups. There were 1224 patients in the high-risk group, which had a shorter median OS than the low-risk group (HR 2.62, 95% CI 2.25–3.04, P < 0.001, Fig. 4A). Further analysis showed that there were no differences between the low-risk groups with and without preRT (HR 1.19, 95% CI 0.92–1.54, P = 0.180, Fig. 4B), but significantly prolonged median OS in the high-risk group who received preRT (HR 0.71, 95% CI 0.56–0.91, P = 0.006, Fig. 4C).

## Discussion

The long-term survival benefit has always been the stumbling block of the radiation-based neoadjuvant treatments for LARC patients, even in the TME era. In this study, we screened 7582 LARC patients including 6066 patients (80.0%) received preRT and 1516 patients (20.0%) received surgery alone from SEER database between 2011 and 2015, and long-term survival benefit of preRT was confirmed in the entire cohort. A nomogram comprising of independent risk factors was constructed to predict the prognosis of LARC patients and further analysis showed that high-risk patients were more likely to benefit from preRT, as indicated by their OS.

Radiotherapy was initially introduced as an adjuvant treatment to prevent local rectal cancer recurrence^14^. Later on, radiotherapy was progressed as a preoperative strategy, resulting in stronger anti-recurrence efficacy, higher R0 resection rate, increased sphincter-preserving, and less radiation toxicity^7,15^. Generally, preoperative radiotherapy, including LCRT and SCRT, followed by instant or delayed surgery, have been the standard treatment for patients with LARC^15,16^. Numerous single-center and multi-center trials have laid the foundation for preRT for LARC management, which is now used in clinics worldwide. In this study, 80.0% of LARC patients received preRT.

However, there has always been controversy regarding preRT. First, the pCR rate following radiation-based neoadjuvant treatments varies different from different modalities (LCRT, 2.2 to 33.8%; SCRT, 0.3 to 28.0%)^5,16–19^. Second, acute radiation-induced injury, such as radiation colitis, decreases treatment compliance and quality of life^20^. Third, late radiation-induced injury, such as fibrosis, increases the surgical dissection difficulty and the risk for postoperative complications^12,20^. Ultimately, the long-term survival benefit (e.g., disease-free survival [DFS] and OS) of radiation-based neoadjuvant treatments (regardless of the modality) is not clear. Since the start of TME, the primary treatment failure indicator is distant metastasis rather than local recurrence, suggesting that the clinical value of preRT for LARC management needs re-evaluation.

The key to correctly evaluate the clinical efficacy of a treatment modality is endpoint selection. pCR is the most widely used endpoint to evaluate neoadjuvant treatments, but using pCR as an optimal surrogate endpoint remains controversial^21^. This may be because the correlation between pCR and long-term survival is not definite^22,23^, and pCR is influenced by other non-treatment factors (e.g., the interval between radiotherapy completion and surgery)^24^. DFS is the time from randomization to recurrence or death from any cause, which was found to be a stronger predictor of OS than pCR among 2795 patients received neoadjuvant treatment^25^. However, DFS is also not an ideal surrogate endpoint because the DFS starting time varies, especially regarding surgery. OS is a hard endpoint for any treatment, although it has been confounded by salvage treatments (in recurrence cases) and potential causes of non-cancer-related mortality. OS also often requires a larger sample size, longer follow-up, and higher costs. The advantages of preRT on the OS were rarely observed among phase III RCTs (Table 4)^6,26–34^, but advantages were identified in a meta-analysis including 6426 trial patients in 2000^35^ and a population-based analysis including 49,439 patients in 2019^13^. The most likely reason for this divergence might be the sample size, in our opinion. In the current study, the long-term survival benefit of preRT was confirmed using population-based analysis of 7582 LARC patients, which confirmed our hypothesis.

**Table 4:** Overall survival in rectal cancer associated with neoadjuvant radiotherapy among phase III RCTs.

Study	Treatment arm (sample size)	Total dose/daily dose (Gy)	Treatment interval time (day)	5-year OS (%)	P-value
VASOG I (1975)^26^	R + S (189)	20/2	14	43.4*	0.042
	S (187)			31.6*	
VASOG II (1986)^27^	R + S (126)	31.5/1.75	Immediate	50.3*	0.997
	S (136)			49.6*	
EORTC (1988)^28^	R + S (166)	34.5/2.3	11	69.1*	0.08
	S (175)			59.1*	
Stockholm I (1990)^29^	R + S (331)	25/5	7	42.0*	NA
	S (348)			41.9*	
Goldberg (1994)^30^	R + S (146)	15/5	7	40.3	0.92
	S (134)			38.8	
Marsh (1994)^31^	R + S (143)	20/5	7	30.1	0.21
	S (141)			30.5	
MRC II (1996)^32^	R + S (139)	40/2	45	31	0.1
	S (140)			38	
Swedish rectal cancer trial (1997)^33^	R + S (573)	25/5	7	58	0.004
	S (574)			48	
Dutch TME trial (2001)^6,10^	R + TME (924)	25/5	< 10 (87%)	62.2	NA
	TME (937)			61.9	

*R* radiotherapy, *S* surgery, *TME* total mesorectal excision, *NA* not applicable, *RCTs* randomized clinical trials.

*Analysis of the results of patients undergoing radical surgery.

In the TME era, local recurrence is no more than a primary cause of treatment failure. Intensified neoadjuvant chemotherapy was also found to be comparable to conventional preRT in terms of OS in the trials of FOWARC^36^ and PRODIGE 23^8^, and even had a weak DFS advantage. Both of above have caused the necessity of preRT to be questioned. In this study, we also constructed a nomogram to identify the potential the long-term survival beneficiary of preRT. According to the current nomogram, preRT was found to bring significant long-term survival benefit only to the high-risk patients rather than low-risk patients. Therefore, we concluded that preRT should been conducted in the management of LARC patients in the era of TME and intensified chemotherapy, especially for those with high-risk score according to the current nomogram.

From the other hand, “watch and wait” strategy has been pouring new vigor into the application of preRT. A retrospective study analyzed 71 patients with a complete clinical response using the “watch and wait” strategy and 21 patients with an incomplete clinical response but a complete pathologic response post-TME^37^. The 5-year OS and DFS rates were 100% and 92% in the “watch and wait” group, and 88% and 83% in the surgery group, respectively. The “watch and wait” advantage was also confirmed in a study by three neighboring UK regional cancer centers with the 3-year rates of non-regrowth DFS (88% vs. 78%), OS (96% vs. 87%), and colostomy-free survival (74% vs. 47%)^38^. pCR was found to be significantly associated with the radiotherapy dose^39^, and the pCR by boosted chemoradiotherapy (60 Gy/30 fractions) was as high as 73% in a prospective single-arm trial^40^. From the other hand, an elevated pCR is also obtained by adding intensified chemotherapy before and/or after preRT in the trials of PRODIGE-23^8^, RAPIDO^5^. Hence, multidisciplinary management combined with patient willingness is strongly recommended to make decision for LARC patients, and intensified curative chemoradiotherapy may be an alternative for select patients^9,41,42^.

In addition, we also found an interesting phenomenon in the present study. Patients over 65 years old were identified to be benefited more from preRT than young ones. Mismatched biology may be the very reason. Young LARC patients are often present with aggressive pathological features, such as elevated CEA levels, poor differentiated grade, which generally response poorly to radiotherapy^43^. From the other hand, cancer stem cell is the cause of radiation resistance, and tumors in young patients are found to have a higher proportion of cancer stem cells^44^. However, this finding needs further exploration.

Nonetheless, this study has several limitations. First, this was a retrospective study. Second, data on circumferential resection and preRT, such as modality, gross tumor volume, clinical target volume, was not available. Third, data on chemotherapy administration were also unknown, which is one of the most important risk factors for long-term prognosis. Hence, we excluded those patients who did not received chemotherapy to decrease the effect of chemotherapy on long-term prognosis. Finally, the TME technique has been popular worldwide since 2002^45^ and we only enrolled patients after 2010, but data on surgery in the SEER database were unavailable.

## Conclusion

preRT could bring long-term survival benefits for LARC patients, especially for those with high-risk score according to the current nomogram. But further validation with a larger sample size and multi-center RCTs with well-designed outcome measurements is also required.

## Supplementary Information

Supplementary Information.

## Data Availability

The datasets analyzed in this study are obtained from SEER database and can be obtained from: https://seer.cancer.gov/data/.
