# Supplementary material for: Identifying the long-term survival beneficiary of preoperative radiotherapy for rectal cancer in the TME era
Source: Sci Rep. 2022 Mar 17;12:4617. doi: 10.1038/s41598-022-08541-1 (PMC8931157; doi:10.1038/s41598-022-08541-1)
Supplement: Supplementary file 1 — Supplementary Information. [file 41598_2022_8541_MOESM1_ESM.pdf]

Supplementary Table S1 Demographic and tumor characteristics of Training Set and Validation set.

| | Training Set, n(%) | Validation set, n(%) | P-value |
|---|---|---|---|
| | N=4549 | N=3033 | |
| Age | | | |
| ≤65 years | 3230 (71.0%) | 2191 (72.2%) | 0.254 |
| >65 years | 1319 (29.0%) | 842 (27.8%) | |
| Sex | | | |
| Male | 2800 (61.6%) | 1849 (61.0%) | 0.623 |
| Female | 1749 (38.4%) | 1184 (39.0%) | |
| Insurance | | | |
| No | 180 (4.0%) | 119 (3.9%) | |
| Yes | 4323 (95.0%) | 2877 (94.9%) | 0.692 |
| Unknown | 46 (1.0%) | 37 (1.2%) | |
| CEA | | | |
| ≤5 ng/mL | 1798 (39.5%) | 1249 (41.2%) | |
| >5 ng/mL | 1381 (30.4%) | 919 (30.3%) | 0.245 |
| Unknown | 1370 (30.1%) | 865 (28.5%) | |
| Stage | | | |
| II | 1644 (36.1%) | 1108 (36.5%) | |
| III | 2897 (63.7%) | 1920 (63.3%) | 0.936 |
| Unknown | 8 (0.2%) | 5 (0.2%) | |
| T stage | | | |
| T0/T1/T2 | 397 (8.7%) | 273 (9.0%) | |
| T3 | 3667 (80.6%) | 2398 (79.1%) | 0.270 |
| T4 | 458 (10.1%) | 346 (11.4%) | |
| Unknown | 27 (0.6%) | 16 (0.5%) | |
| N stage | | | |
| N0 | 1591 (35.0%) | 1080 (35.6%) | |
| N1 | 2270 (49.9%) | 1517 (50.0%) | 0.825 |
| N2 | 680 (14.9%) | 431 (14.2%) | |
| Unknown | 8 (0.2%) | 5 (0.2%) | |
| Tumor differentiation | | | |
| Grade I/II | 3547 (78.0%) | 2414 (79.5%) | |
| Grade III/IV | 516 (11.3%) | 311 (10.3%) | 0.215 |
| Unknown | 486 (10.7%) | 308 (10.2%) | |
| Tumor size | | | |
| ≤3 cm | 1097 (24.1%) | 714 (23.6%) | |
| 3-5 cm | 1529 (33.6%) | 1041 (34.3%) | 0.605 |
| >5 cm | 1319 (29.0%) | 901 (29.7%) | |
| Unknown | 604 (13.3%) | 377 (12.4%) | |
| Tumor deposits | | | |
| Negative | 3656 (80.4%) | 2438 (80.4%) | |
| Positive | 577 (12.7%) | 384 (12.6%) | 0.999 |
| Unknown | 316 (6.9%) | 211 (7.0%) | |
| Perineural invasion | | | |
| Absent | 3508 (77.1%) | 2368 (78.1%) | |
| Present | 553 (12.2%) | 350 (11.5%) | 0.607 |
| Unknown | 488 (10.7%) | 315 (10.4%) | |

(continued on next page)

Table S1 (Continued)

| | Training Set, n(%) | Validation set, n(%) | P-value |
|---|---|---|---|
| | N=4549 | N=3033 | |
| Number of LND | | | |
| <12 | 1203 (26.4%) | 789 (26.0%) | |
| ≥12 | 3339 (73.4%) | 2238 (73.8%) | 0.831 |
| Unknown | 7 (0.2%) | 6 (0.2%) | |
| Preoperative radiotherapy | | | |
| No | 890 (19.6%) | 626 (20.6%) | 0.264 |
| Yes | 3659 (80.4%) | 2407 (79.4%) | |

CEA, carcinoembryonic antigen; LND, lymph node dissection

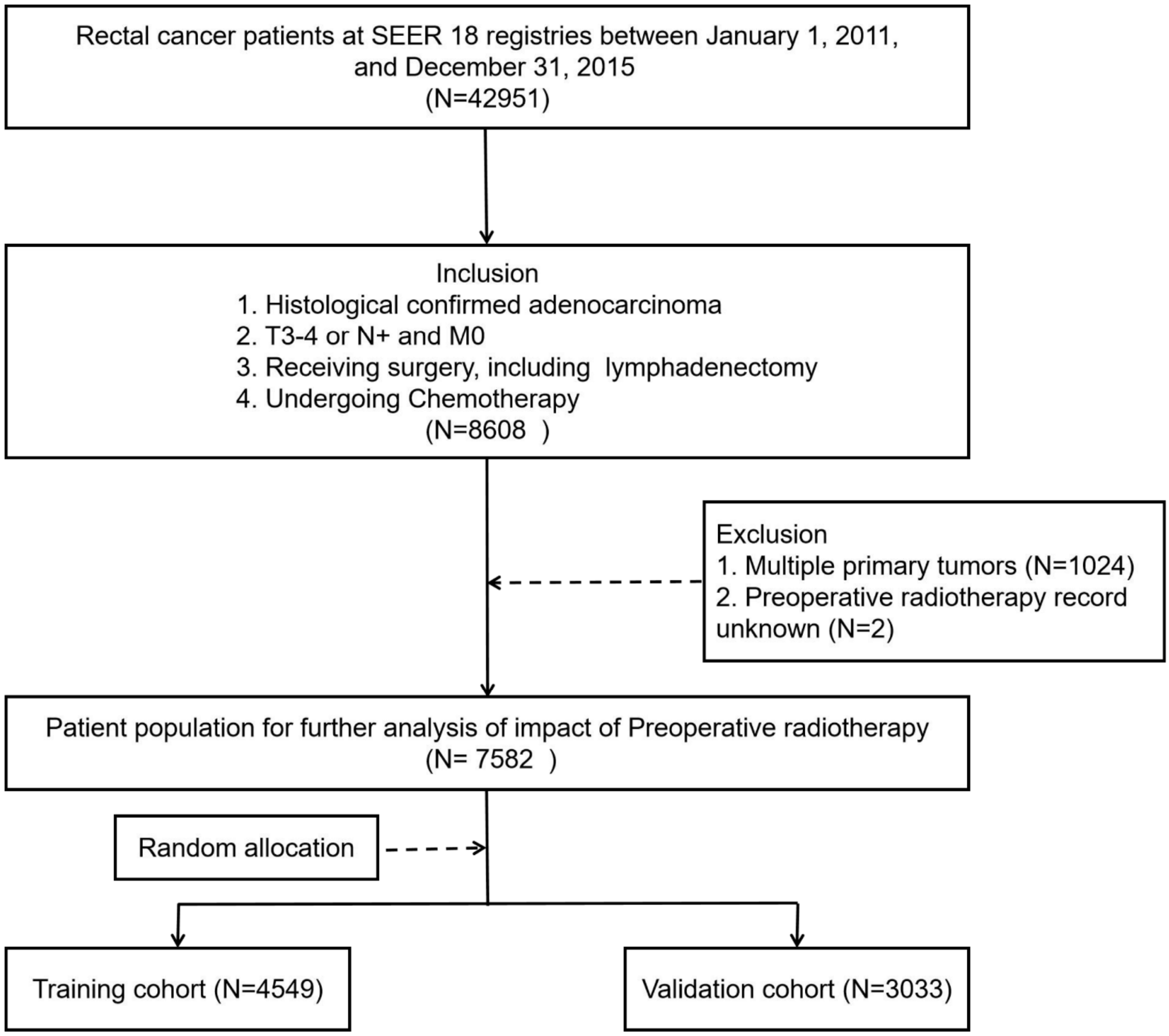


Supplementary Figure S1 Flow diagram of patient population selected from SEER database.

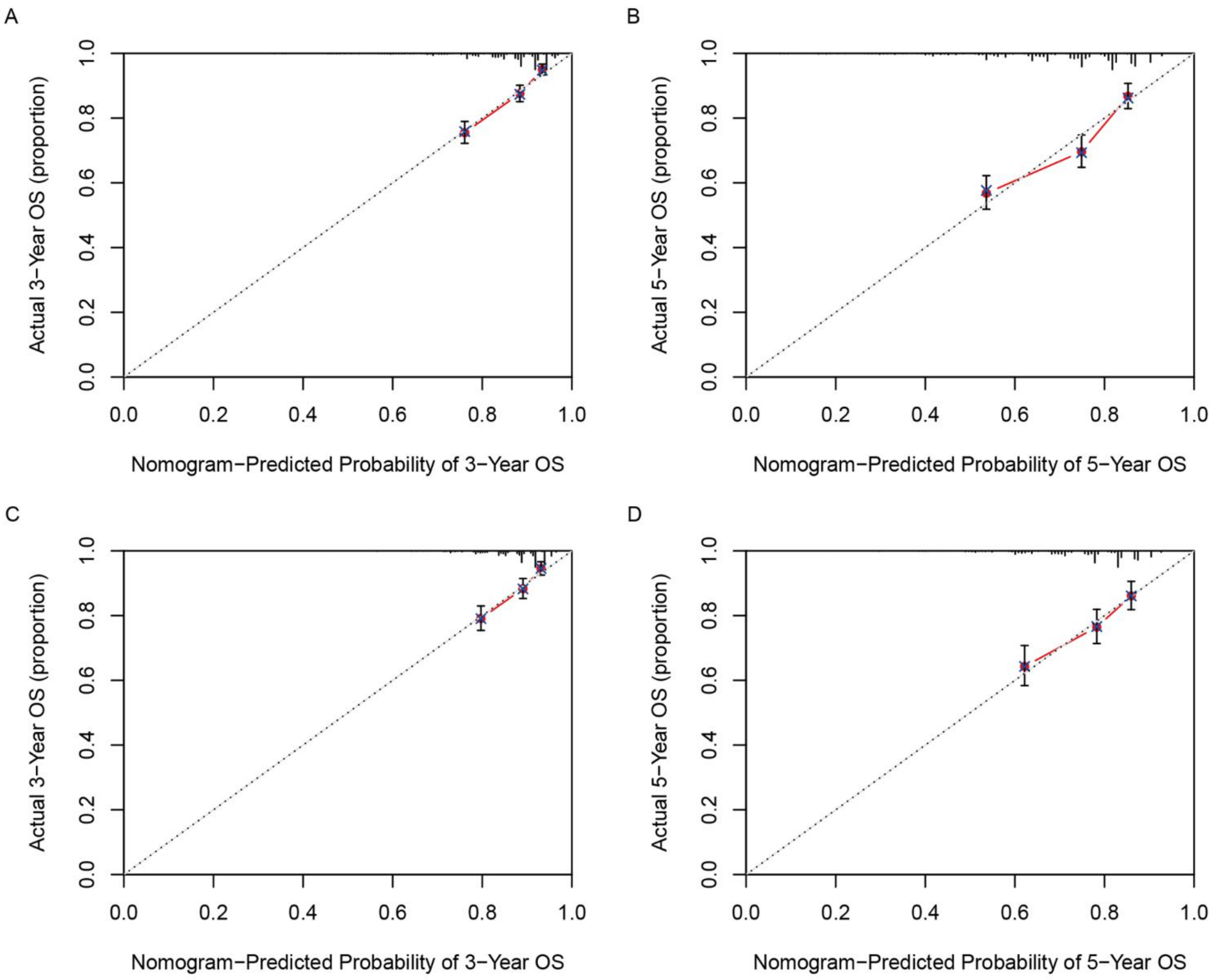


Supplementary Figure S2 Calibrations of the nomogram for predicting survival rates. (A) 3-year, (B) 5-year in the training set and (C) 3-year, (D) 5-year in the validation set.

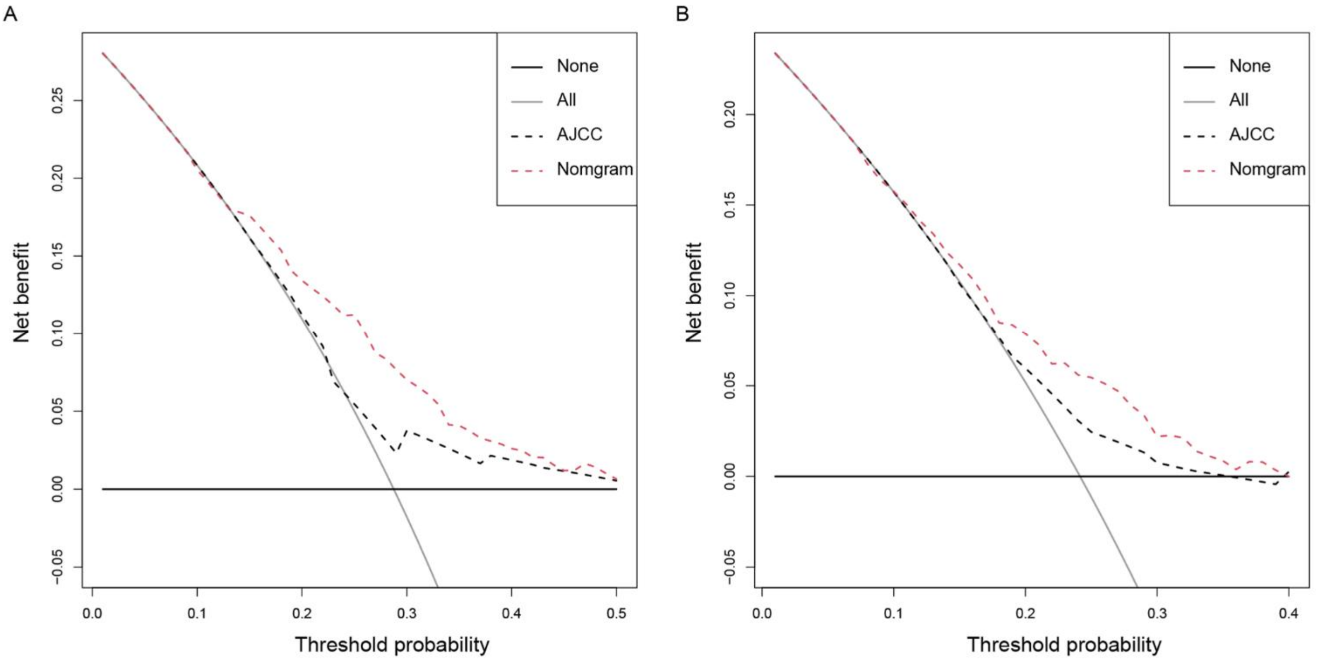


Supplementary Figure S3 Decision curve analysis (DCA) of overall survival using nomogram and the 8th American Joint Committee on Cancer staging system. (A) DCA of 5-year OS in the training set; (B) DCA of 5-year OS in the validation set.
